# Are There Sensitive Periods for Food Acceptance in Infancy?

**DOI:** 10.1007/s13668-017-0203-0

**Published:** 2017-04-29

**Authors:** Gillian Harris, Sarah Mason

**Affiliations:** School of Psychology, University of Birmingham, Birmingham, UK

**Keywords:** Sensitive periods, Critical periods, Infancy, Childhood, Feeding eating, Neophobia, Disgust, Sensory hypersensitivity, Tactile defensiveness, Oral motor function

## Abstract

**Purpose of Review:**

A sensitive period in development is one in which it is easier for learning to take place; the behaviour can however still be learned at a later stage, but with more difficulty. This is in contrast to a critical period, a time at which a behaviour must be learned, and if this window of opportunity is missed, then the behaviour can never be acquired. Both might determine food acceptance in childhood.

**Recent Findings:**

There is evidence to support the idea of a sensitive period for the introduction of tastes, a critical period for the introduction of textures and for the development of oral motor function, and a possible critical period for the introduction of new foods but only in children where there is an innate disposition to develop early and extreme disgust responses.

**Summary:**

There are both sensitive and critical periods in the acquisition of food preferences.

## Introduction: Sensitive Periods and Critical Periods in Development

Firstly, what do we mean by a ‘sensitive period’, and how does this differ from a critical period in development? A sensitive period is a limited period during which the brain is particularly receptive to the effects of experience. Such periods ‘allow experience to instruct neural circuits to process or represent information in a way that is adaptive for the individual’ [1 p91]. After a sensitive period, learning may still occur but ‘extra energy’ is required for this learning to take place [1]. When this period provides experience essential for normal development ‘which alters performance permanently’, then this is described as a critical period [2], and the brain then becomes resistant to subsequent change [1]. However, there is now some evidence that even processes not attained during a critical period can be attained in adulthood [3]. Critical and sensitive periods both need an age of onset and an age or stage at which learning can no longer occur or occur with such ease.

We must also ask ourselves what might be the function of a sensitive, or even a critical period, for development in infancy, and the relevance of these stages to food acceptance. Given that all mammals must be open to learning about changes in their environment and changes in their role within a social group as they age, why would a crucial period for learning new skills be of advantage to a species? The answer must lie in the way in which the brain develops, with neural networks set up in the young to facilitate necessary learned behaviours as other unnecessary pathways die off [3]. However, there are very few critical periods for learning in infancy in which this occurs; the most cited one of these is within the visual system, that of acquired stereoscopic vision [4].

Critical periods are not seemingly advantageous and relate to the manner in which neural circuits develop, but why might sensitive periods be advantageous? This is because there are crucial periods during which the infant interacts with the environment; this interaction is determined by intrinsic factors, and the infant is primed to learn about stimuli that will facilitate development and contribute to subsequent survival. Learning about which foods to eat might therefore be subject to the ‘sensitive’ attribute. As human infants progressively need to eat food, then they need to learn very quickly which foods are safe, which foods are eaten by those around them, and their taste preferences need to be modified accordingly. Food acceptance per se cannot be hard wired given the wide range of environments inhabited by the human race and the wide range of foods which provide the diet of different cultural groups. In the beginning, all food preferences need to be learned. The presence of the timed ‘off switch’, which we will see does occur but not until late infancy, might be advantageous in that it directs the infant away from non-foods, once the initial learning has taken place and the infant is able to forage. This ‘off switch’ may, however, not always herald the end of availability of appropriate neural pathways but rather the presence of competing behaviours. Early research studies looking at isolation studies carried out on macaques seemed to show that there were critical periods during which social interaction skills had to develop. However, subsequent research [5] demonstrated that it was not that the infant macaque could not interact with age mates when introduced to them after periods of isolation, but that other fear behaviours interfered to prevent this interaction. So it could be that the limiter in our possible critical period is a fear response and that with ‘extra effort’, this response might be overcome.

There are three points of development and domains at which this easy acceptance or later refusal might occur in relation to feeding and eating behaviour: taste and smell, tactile stimulation and texture, and visual appearance. The mechanisms which are needed to sense taste and smell are more or less completely developed by birth and so acceptance post-natally in each of these domains [6] is related to exposure, with additional learned oral motor skills, and the development of the distaste-disgust response.

## Taste

Infants are exposed to flavours in utero and via breast milk. However, the flavours to which the infant is exposed by these routes can vary widely from mother to mother and from feed to feed [7, 8], and although they might slightly prime the infant for subsequent acceptance of new foods, the advantages which they confer do not outweigh the mere introduction of tastes during the period of the introduction of complementary foods [9].

The infant is born with a preference for a sweet taste, and this may well be beneficial in that it directs the infant to ingest foods that might be a source of energy, and to reject water. The taste of bitter, however, is rejected at birth [10, 11] because of the possible association between a bitter taste and toxicity. All other taste preferences are subsequently learned through exposure [9, 10]. Early work carried out on the acceptance of tastes in food showed that there was an easy acceptance for a salt taste if this was added to a tasteless rice base and that this acceptance was dependent upon number of exposures rather than the amount eaten [12, 13]. A study carried out on a small sample of infants tested (15–26 weeks), for their preference for a tastant (salt) in the first food fed to them, cereal [14], additionally showed that intake for the food with the added tastant was significantly better for the younger infants (16–17 versus 18–25 weeks). However, Johnson [15] looked at the acceptance of tastants added to rice-based cereal. Infants were given a teaspoonful of a different tastant for 14 days in the first food fed to them (mean infant age 14 weeks). There was a clear and fast exposure effect for sour and salted rice; infants learned to like these flavours quickly and easily. However, by chance, the infants allocated to the condition in which they were given a bitter tastant added to cereal were slightly older (mean age 17 weeks) than were those exposed to the salt and sour tastes. For those infants given bitter tasting cereal, there was no exposure effect; those infants fed the bitter tasting cereal preferred the taste-free rice-based cereal. This effect might have been due to the bitter taste or due to the slightly later age of introduction, an effect noticed in infants fed bitter tasting (proteinhydrolysate) formulae [16]. Infants younger than 3–4 months would readily accept strong tasting bitter formulae; however, infants over 5–6 months of age refused them. In contrast, infants who had had experience with the taste prior to 5–6 months would accept the strong tasting formulae at the later age [17]. This easy acceptance of a bitter tasting formula after early exposure continues into later childhood [18]. The taste preference learning was also exactly specific to the formula experienced [19]. Therefore, what was learned in this early sensitive period was that flavours initially linked with possible toxicity, if paired with good nutritional outcome, could be accepted. Anecdotally, many parents report that it is difficult to change their infant from any formula in later infancy once they have learned to accept a specific formula taste in early infancy.

There was some generalization noted from this early acceptance of a bitter tasting formula to the acceptance of bitter tasting food. The infants given bitter hydrolysates preferred sour-flavoured juices at 4–5 years [20]. But such taste preferences do not seem to generalize from liquid to solid food. Infants fed protein hydrolysates did not prefer broccoli, which is bitter, to carrots which are sweet [21].

## First Foods

The early and easy acceptance of all new foods, rather than mere tastes, at a slightly younger age in the period of the introduction of complementary foods (4–6 months) has been observed in many studies [22••, 23]. It has been shown that the earlier fruit and vegetables were introduced in the infant’s diet, the better their acceptance, both in infancy and at a later age in childhood [24–30]. This possibly reflects the difficult nature in terms of texture and tastes shared by many fruit and vegetables, both properties which children find aversive [6, 31••]. It would seem therefore that early experience with both difficult tastes and textures has a facilitating effect. However, it should also be remembered that those introduced early, and to a variety of fruit and vegetables, probably live in an environment which generally facilitates the acceptance of a wider diet.

There is also a generalization effect with all new foods and tastes. The more new tastes that are introduced early (4–5 months as opposed to 6 months) then the better the acceptance of a new food [32]. In addition, those infants fed home prepared foods possibly featuring a wider range of taste and textures were more likely to eat a range of fruit and vegetables at 7 years, than were those fed commercially produced foods [33]. Difficult tastes are therefore not only better accepted if introduced earlier to the infant but also if they are introduced frequently [34]. It may also be advantageous if the infant experiences a continuation from taste to texture to appearance; that is they learn to recognize the food in all of its different presentations, as may well be the case with home prepared rather than with commercially produced introductory foods.

Two studies of interest which add to this literature have been carried out with premature infants. It was observed that premature infants not only thrive better with the earlier introduction of complementary foods [22••, 35] but also more readily accepted novel foods than did age matched full-term infants (5–6 months) [22••]. In this latter study, the mean gestational age of the premature infants was 30.6 weeks and that of the term infants was 39.1 weeks; at the point of testing, the age of the premature infants was not corrected for gestational age. The authors hypothesized that the premature infants would display more negative emotion when exposed to novel foods than would the full-term infants, but analysis of facial expression in response to the introduction of new foods showed a more positive acceptance by the pre-term infants. The pre-term infants were essentially 2 months younger than were the full-term infants.

Although some new tastes quite often need more than one exposure, especially if the taste has not been experienced before [36], acceptance does occur, in general, quite easily and seemingly faster at a younger age [22••] when the infant is offered frequent small tastes of the food eaten by those around them. The acceptance of a bitter taste is, however, more problematic; this is a taste often associated with toxicity and is therefore a taste to which it is appropriate for the infant to react and possibly subsequently reject. However, if the taste is associated in very early infancy with the only source of good nutrition, then this taste will be accepted at the earlier age, but not at the later age when other sources of good nutrition have been experienced. To a great extent, then the acceptance of food tastes is easier at an earlier age but not totally dependent upon developmental stage. Tastes are learned in context and gradually associated with foods and food textures [37] which become increasing more complex. Unless the infant (and child) has innate taste aversions, such as an extreme response to bitter [6, 38], then tastes can be accepted and modified throughout life [39]. However, in an adult, the motivation to try a new taste may be the defining characteristic determining whether or not that taste or taste modification is accepted. And even in some children who have had foods withheld in the first year of life because of infantile milk allergies, such foods may well be refused in later childhood [40•].

One further innate characteristic also acts as a limiter on how many tastes an infant or child is willing to try, that of sensory hypersensitivity, a trait linked to both food ‘fussiness’, and a refusal to try new foods [41, 42]. One study has recently shown that the age of introduction of new foods in the 4–6-month introductory period is more salient for those infants rated higher on tactile hypersensitivity. Hypersensitive infants introduced to foods earlier within the introductory period were more likely to accept a new food than were infants introduced later (4–5 months rather than 5–6 months) [43].

## Accepting Texture and Learning the Appropriate Oral Motor Skills

The concept of a sensitive period for the introduction of solid food was first suggested by Illingworth [44 p840,] based on case studies of hospitalized infants.

‘Children should be given solids to chew at a time when they are developmentally ready: in an average child, this age is 6 to 7 months. If they are not given solids then (as distinct from thickened feedings, which can be given any time after birth), they are very apt to be difficult about taking them later, failing to chew, refusing the solids, or vomiting. Failure to give solids may be due to mismanagement in normal children, including over anxiety about their choking’.

There is evidence to support this suggestion that the early introduction of textures, as soon as possible after 6 months and certainly before 12 months, is essential.

A new born infant is born with a disposition to ingest, with sucking being the earliest means of receiving nutrition [45]. Early nutritive sucking comprises forward and backward movements of the tongue and is supported by both physiological and anatomical factors: rooting and sucking reflexes, the lips, tongue, and jaw acting as a single unit; the size of the oral cavity; sucking pads; and proximity of oral and laryngeal structures [45–47]. It is generally reported that premature infants cannot suck nutritively until the age of 34 weeks gestation but non-nutritive sucking is reported earlier [46, 48]. The difference between nutritive and non-nutritive sucking is that nutritive sucking requires the coordination of bursts of suck-swallow-breathe cycles whereas non-nutritive sucking is a burst of sucking and breathing with intermittent swallows. Reviews of the literature on non-nutritive sucking have shown that the promotion of non-nutritive sucking in premature babies who require tube feeding, usually by the use of pacifiers, leads to a quicker transition to oral feeding and shorter stays in neonatal units [49–51]. While the benefits of non-nutritive sucking on the premature population are well researched, it is less clear whether these benefits continue in older infants who are unable to take nutrition orally during the early months of life. Mizano and Ueda [52] noted that, in their experience, once a baby reached 6 months or more, it was very difficult to establish bottle feeding for the first time.

One factor that may be involved in the difficulty of introducing breast or bottle feeding after an interval of non-oral feeding is that the infant reflexes of rooting and sucking diminish over the first 4 months of life, an example of a critical period of learning. At first, sucking will be triggered automatically by any sort of stimulation of the lips and tongue but gradually, this reflex response comes under voluntary control. Morris and Klein [47] described how the baby develops separation of movement that enables the jaw, lips, and tongue to move separately and thus perform more complex oral motor tasks, an extension to the reflex suck and swallow.

The attainment of early oral motor skills needed to process more solid foods around the mouth shows a wide diversity across individuals. Carruth and Skinner [53] found that the mean age at which infants used the tongue to move food to the back of the tongue to swallow was 4.95 months, with a standard deviation of 1.27 and a range of 2.0–7.5 months. This variation may reflect normal differences in the development of the central nervous system and opportunities the infant has to practice skills [53, 54]. Weaning guidelines currently recommend that spoon feeding should not be commenced before 17 weeks of age but infants started taking solids from a spoon at this earlier age in previous decades [55], so the oral motor skills required to eat from a spoon precede the need for their use.

The late introduction of textured solids that is foods that need to be processed with a side to side tongue movement is associated with poorer acceptance of these foods, and it has been suggested that there is at least a sensitive period for the acquisition of chewing skills [44]. Northstone, Emmett, and Nethersole [56] found that the introduction of lumpy foods at or later than10 months was associated with more feeding difficulties at 15 months of age. These feeding difficulties and a poorer acceptance of fruit and vegetables (foods of complex texture) were also observed in these children at the later age of 7 years [57]. The studies were, however, reported observations and other factors could have contributed to the later feeding difficulties.

Mature chewing is a complex combination of movements of the lips, tongue, and jaw, and fully mature chewing can take several years to develop [58, 59]. It begins with a mixture of sucking, biting, and up/down ‘munching’ movements, characterized by regular patterns of reciprocal activation between antagonistic muscle pairs [58]. At the same time, the tongue begins to move laterally, from centre to side. Wickendon [48] described how this munching enables a baby to manage foods such as infant rusks, breadsticks, and toast, from about 6–7 months. A study of the development of chewing in children aged 6 months to 2 years found that while chewing efficiency increased over the entire period between 6 months and 2 years, the most marked changes occurred between 6 and 10 months [60]. It has been reported that infants as young as 7 months were able to adjust their jaw movements in response to the different consistencies of food [58, 59] and that the best predictor of acceptance of chopped carrots in 12-month-old infants was shown to be the infant’s previous experience with carrot pieces [61].

The poor acceptance of textured foods by children who are introduced to them after the first year of life would appear to have at least two components: firstly, a refusal to try unfamiliar foods as the child moves into the neophobic stage of food refusal in the second year of life and, secondly, an inability to manage the texture because chewing skills have not been acquired through practice [62]. Most researchers [63] agree that movement patterns, such as lateral tongue movements, are texture dependent and therefore do not emerge unless the child is given the particular textures requiring these skills.

Another component in the acceptance of textured foods is that the side to side processing of food within the mouth serves to desensitize the inner sides of the cheeks. Children who are introduced later than usual to textured foods often attempt to process these foods using a ‘liquid swallow’, in which foods are not processed at the sides of the mouth but are swallowed directly over the back of the tongue. Lumpy textured food which lands onto the back of the tongue provokes a gag and retch reflex, which is aversive to the child. In addition to this, children who do not process foods with a side to side tongue movement which positions food appropriately for processing become hypersensitive to any tactile stimulation and therefore find the experience of foods at the sides of the mouth aversive [64, 65]. Parents may also respond to this gag response by further delaying the introduction of solid-textured foods [66]. Sensory hypersensitivity may therefore, once again, be a factor in the salience of the ‘sensitive’ period, this time in the introduction of textured foods. Sensory-hypersensitive children are reluctant to allow food to go into the sides of the mouth and also reluctant to process the more difficult textures. Higher tactile sensitivity is particularly associated with rejection of foods of difficult texture [41, 65].

## Appearance: the Neophobic Stage

Infants learn to accept the taste and then the texture of foods that are fed to them and that they learn to perceive as ‘safe’ foods. However, before they get to the stage of foraging, in their second year, they need to know what their safe foods look like and need to attend quite closely to the detail of the appearance of that food.

The neophobic stage, as it is defined for humans, describes a response of refusing food on sight [66]. The way that the food looks or the way in which the food is packaged predicts the safety of the food, and any deviation in the smallest detail will lead to this rejection [67]. Even foods that have been accepted prior to the onset of the neophobic stage might be refused if their appearance changes on subsequent presentations [68]. This stage is described as starting at around 20 months [69] and continuing until around 6 years [70], by which time the response has gradually diminished. The strength of the neophobic response is also related to the child’s degree of sensory hypersensitivity [41] and to some extent genetically determined [71, 72]. The onset of this stage also occurs at the time at which infants are showing contamination fears and therefore an early form of disgust [73–75]. A food that is liked might be refused if touched by a food that is disliked. Although it is relatively easy to get acceptance of a new taste or new food when that food has not previously been experienced, where there is motivation to try the food and where others are modelling the food [76–78], once a food has been deemed disgusting it would seem that it is almost impossible to get that food accepted into the diet [79, 80]. Even where new foods are tried, it takes more exposures to gain an acceptance of those foods in later infancy and childhood than it does in early infancy [22••, 81].

Those children who are sensory hypersensitive are more likely to show strong neophobic responses and extreme disgust responses. In the extreme, some children form a strong disgust response to all new foods or variants of known foods [82]. In these children, the fear of new foods can remain until adulthood; new foods can evoke a disgust and fear response and texture refusal can worsen as the child becomes more orally defensive [64, 83].

This may be an example, not of a critical period however, but of interfering or competing behaviours. The formation of a disgust response towards foods that have not been accepted during the neophobic stage, perhaps necessary in early humans to prevent the ingestion of non-foods, blocks learning about new food tastes and textures. When no disgust or fear response has formed, then older children and adults can be motivated to try new foods that they deem safe to eat. The defining factor here is the sensory hypersensitivity of the child; the experience of the food interacts with the innate disposition of the child to produce an extreme fear response and it is not that neural pathways can no longer form to promote food acceptance.

## Summary

It is easier to get new tastes accepted from birth and new complex flavours accepted in the early months of the introduction of complementary foods, between 4 and 6 months. However, acceptance of new tastes can be learned throughout life if the older child or adult is motivated to accept the repeated tastings that are required, not only for acceptance but also for pleasurable anticipation of the taste. For some of course, those who are genetically programmed to dislike certain tastes such as bitter [38] or those who are sensory hypersensitive [84], then simply getting older will not change acceptance; there will be no increased motivation to try. The factor of motivation is, however, an important one. Early childhood food preferences and experience predict later childhood and adult food preferences and dietary range [33, 85, 86]. So the sensitive period here is one of setting up habitual patterns of eating which may or may not be subject to later lifetime change. Of greater significance is that of acceptance of texture and the oral motor skills which develop in response to tactile stimulation, and this does have features of a sensitive, or even critical, period with a clear ‘off switch’. The response to texture may well be a function of innate differences, in that sensory-hypersensitive infants are less likely to accept different textures with ease [65, 87], but it can also be delayed by lack of early experience. Those infants who have not experienced textured foods in the first year of life find such foods difficult to accept and to process within the mouth. Similarly, in some, the development of the disgust response to specific foods is also a heightened innate response. In others, however, it could be due to a lack of learning via exposure [40•] in that foods that might be seen to be inherently disgusting in the toddler period could well have been accepted had they been introduced in the early period of complementary food introduction. One example of this response is that of seal blubber acceptance in the Inuit child, a food which would not be accepted or even approached without an extreme disgust response in those reared in other environments. The onset of disgust in the second year of life does therefore have features of a critical period of food acceptance, as well as that of the acceptance of textured foods and the development of oral motor skills.
